# Circular RNA circSMARCA5 is a prognostic biomarker in patients with malignant tumor: a meta-analysis

**DOI:** 10.1186/s12885-021-08316-3

**Published:** 2021-05-25

**Authors:** Fan Chao, Shiyu Wang, Cong Zhang, Dunsheng Han, Zhe Ma, Gang Chen

**Affiliations:** Department of Urology, Jinshan Hospital, Fudan University, Shanghai, 201508 China

**Keywords:** Biomarker, Circular RNA, circSMARCA5, Malignant tumor

## Abstract

**Background:**

Malignant tumor is one of the most serious diseases endangering human health. Circular RNAs play an important role in the tumorigenesis and progression of various malignant tumors. Although various studies have investigated the biological function of circular RNA circSMARCA5 in malignant tumors, the prognostic value of circSMARCA5 in malignant tumor patients has not been systematically analyzed.

**Methods:**

Relevant studies were obtained from the PubMed and Web of Science database. The quality of the enrolled studies was evaluated using the Newcastle-Ottawa Scale quality assessment system. Survival features and clinicopathological features were assessed using pooled hazard ratios and odds ratios with 95% confidence intervals, respectively.

**Results:**

Overall, 7 relevant publications were enrolled in the meta-analysis. CircSMARCA5 expression was significantly correlated with better OS (HR = 0.51, 95%CI 0.41–0.65) or DFS/RFS/PFS (HR = 0.56, 95%CI 0.43–0.73) in malignant tumors. In the pooled analyses of clinicopathological characteristics, malignant tumors with higher circSMARCA5 were better differentiated (OR = 0.41, 95%CI 0.19–0.88). CircSMARCA5 expression was correlated with less advanced TNM stage (OR = 0.33, 95%CI 0.19–0.55). Moreover, malignant tumors with higher circSMARCA5 expression have less advanced lymph node metastasis (OR = 0.26, 95%CI 0.08–0.79).

**Conclusion:**

These results indicated that circSMARCA5 was a promising biomarker in malignant tumors, which may potentially facilitate clinical decisions in the future.

**Supplementary Information:**

The online version contains supplementary material available at 10.1186/s12885-021-08316-3.

## Background

Malignant tumor is one of the most serious diseases endangering human health [1]. America Cancer Society estimated 1,898,160 new cancer cases and 608,570 deaths in 2020 in the United States [2]. A number of studies have confirmed that circular RNAs (circRNAs) are involved in the metastasis [3, 4], angiogenesis [5], proliferation [6], and drug resistance [7] of malignant tumors. Furthermore, circRNAs can be used as diagnostic and prognostic markers for malignant tumors [8, 9]. Previous meta-analyses have verified the prognostic values of ciRS-7 [10] and circHIPK3 [11] in malignant tumor patients.

Circular RNA circSMARCA5 is derived from the 15th and 16th exon of SWI/SNF related, matrix associated, actin dependent regulator of chromatin, subfamily a, member 5 (SMARCA5) gene (circBase [12] ID: hsa_circ_0001445) and located at chr4: 144464662–144,465,125. CircSMARCA5 was downregulated in various malignant tumors, and could serve as prognostic biomarker of non-small cell lung cancer [13], intrahepatic cholangiocarcinoma [14], hepatocellular carcinoma [8], gastric cancer [15], glioblastoma [16], multiple myeloma [17] and colorectal cancer [18]. Yu et al [8] reported that downregulation of circSMARCA5 was correlated with aggressive characteristics in hepatocellular carcinoma and served as an independent risk factor of survival. CircSMARCA5 inhibited growth and metastasis of hepatocellular carcinoma through miR-17-3p/miR-181b-5p-TIMP3 pathway. Barbagallo et al [16] claimed that circSMARCA5 expression was correlated with overall and progression free survival of glioblastoma multiforme patients.

Although various studies have suggested the biological functions of circSMARCA5 in malignant tumors, the prognostic value of circSMARCA5 in malignant tumor has not been systematically analyzed. Thus, we designed the present systematic review and meta-analysis to summarize the prognostic value of circSMARCA5 in malignant tumors.

## Methods

### Search strategy

The PubMed, Web of Science, Cochrane Library, and Embase databases were searched for eligible studies until April 21, 2021 on circSMARCA5. The following key words were used for searching all the studies related to circSMARCA5: (SMARCA5 OR circSMARCA5 OR hsa_circ_0001445) AND (circRNA OR circular RNA OR “RNA, Circular”[Mesh]). Reference list of the reviewed articles were reviewed for potential eligible publications.

### Inclusion and exclusion criteria

Two independent reviewers (Fan Chao and Shiyu Wang) evaluated the studies independently. Any discrepancy was resolved by the supervisor (Gang Chen). The inclusion criteria were as follows: (i) The expression levels of circSMARCA5 were determined in any human malignant tumors; (ii) Studies in which patients were stratified by the expression levels of circSMARCA5; (iii) The correlation between circSMARCA5 expression and prognosis of patients with malignant tumors was determined. The exclusion criteria were as follows: (i) Did not meet the inclusion criteria; (ii) Reviews, comments, letters, or case reports; (iii) Studies that only investigated the biological function of circSMARCA5.

### Data extraction and quality assessment

Two reviewers (Fan Chao and Shiyu Wang) extracted the data from the identified publications independently. The following data were extracted from each publication: name of the first author, year of publication, country, region, cancer type, study design, number of cases, follow-up time, detection method of circSMARCA5, survival outcome, hazard ratios (HRs) of circSMARCA5 for survival, and correlation between circSMARCA5 expression and clinicopathological characteristics. The Newcastle-Ottawa Score (NOS) quality assessment system [19] was used to determine the quality of enrolled studies. Enrolled studies were scored according to case definition, representativeness of the cases, selection controls, definition of the controls, comparability of cases and controls, ascertainment of exposure, same method of ascertainment for cases and controls, and non-response rate. Studies with a score ≥ 7 were considered high quality. Results were visualized using Review Manager 5.4 software.

### Statistical analysis

The meta-analyses were conducted using Stata 15.1 software (Stata Corporation, College Station, TX, USA). The correlation between circSMARCA5 expression and prognosis of malignant tumor patients were analyzed using the pooled HRs and 95% confidence interval (CI). The pooled HRs and 95% CI were extracted from the Kaplan-Meier curves if they were not reported in the publication using Engauge Digitizer software (https://github.com/markummitchell/engauge-digitizer) and reported methods [20]. The association between circSMARCA5 expression and clinicopathological characteristics were analyzed using pooled odds ratios (ORs) and 95% CI. Higgins *I*^*2*^ statistics [21] were used to analyze the heterogeneity. Sensitive analysis was conducted by omitting the studies one by one in order to identify the effect of an individual study on the pooled HRs or ORs.

## Results

### Results of the literature search

**Fig. 1:**
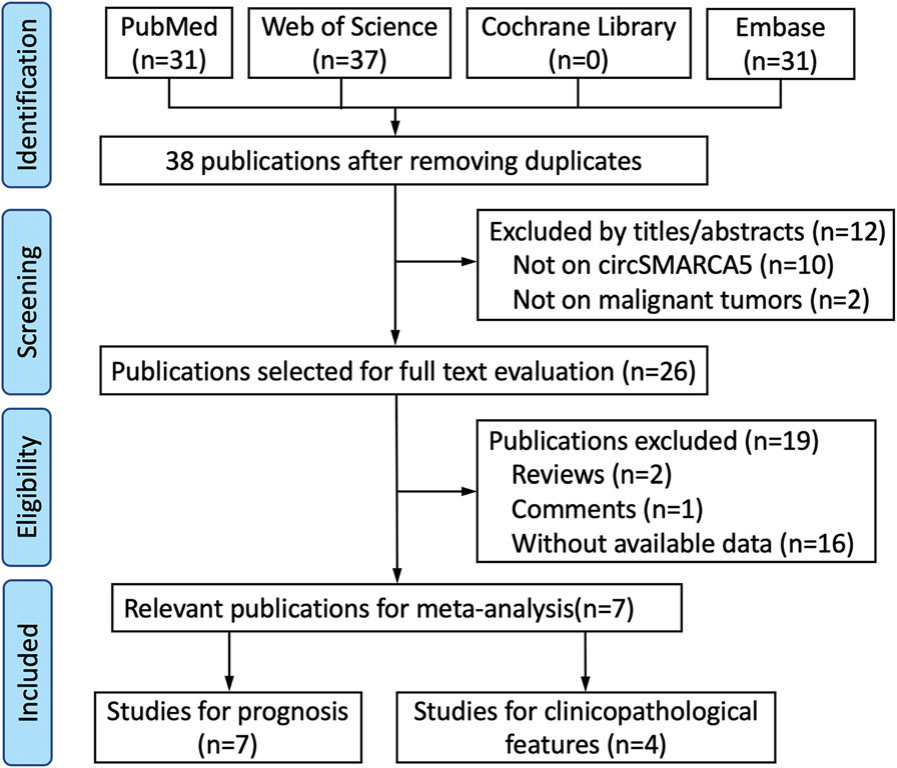
Flow chart of screening eligible articles for the meta-analysis

We retrieved 99 publications from the databases mentioned above and 38 publications after removing duplicates. Twelve publications were excluded by reviewing titles and/or abstracts. After reviewing the full text, we excluded 2 reviews, 1 comment, and 16 publications without available data. Finally, 7 relevant publications were enrolled in the meta-analysis. A flow chart of screening eligible articles for the meta-analysis was shown in Fig. 1.

### Characteristics of the enrolled studies

**Fig. 2:**
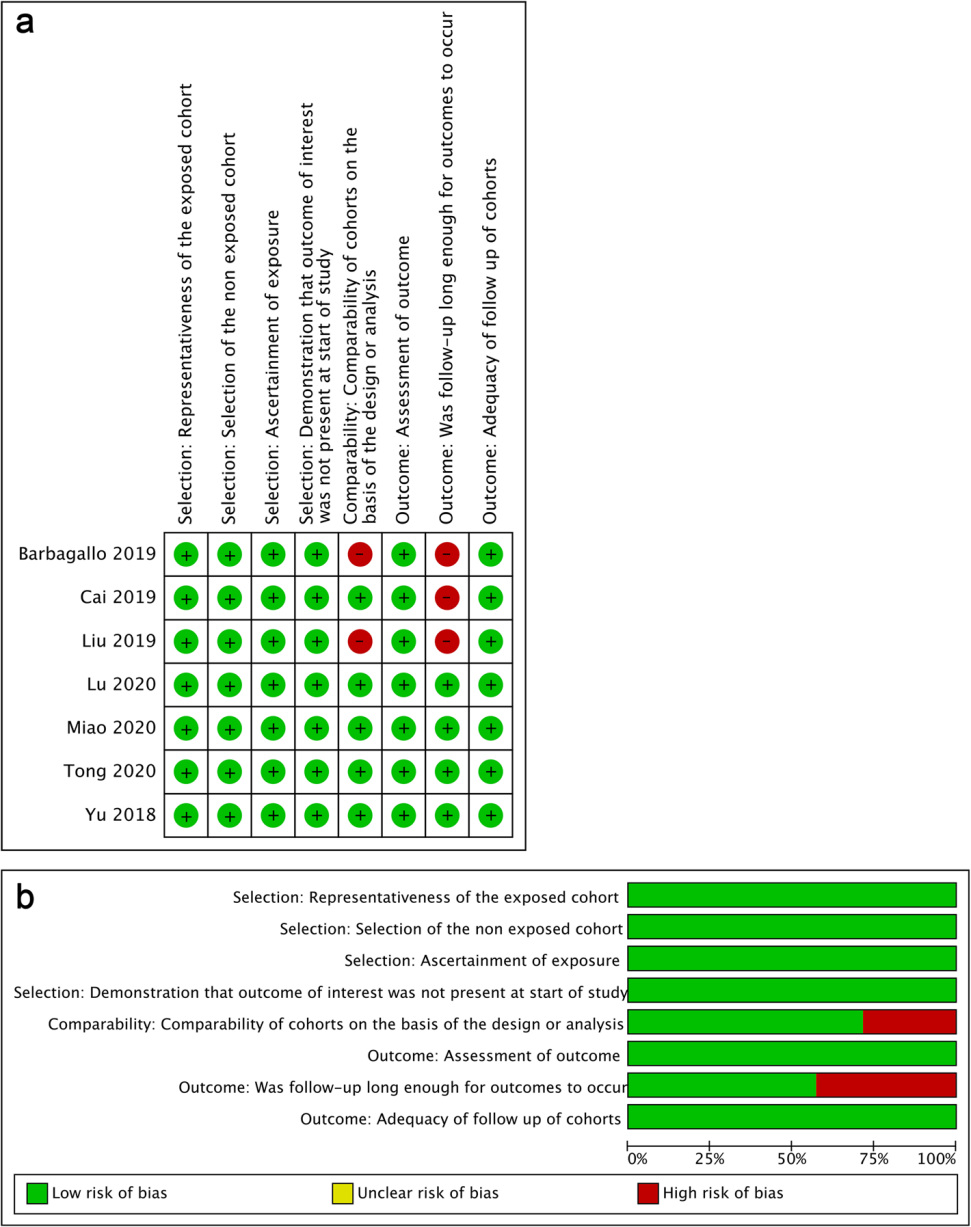
Quality assessment of the enrolled studies. **a** Each risk of bias item for each included study. **b** Each risk of bias item presented as percentages across all included studies

The enrolled studies were published from 2018 to 2021. Seven types of malignant tumors were included: glioblastoma, gastric cancer, intrahepatic cholangiocarcinoma, non-small cell lung carcinoma, multiple myeloma and colorectal cancer. All enrolled studies detected the expression of circSMARCA5 using real-time quantitive polymerase chain reaction (RT-qPCR). Relative expressions of circSMARCA5 were low in all types of malignant tumors. The characteristics of the enrolled studies were summarized in Table 1. The quality of the enrolled studies was estimated by NOS. Most studies had high methodological quality (Fig. 2a), whereas a few studies had deficiencies in comparability and follow-up time (Fig. 2b). All the enrolled studies were prospective studies.

**Table 1:** Characteristics of the studies included in the present meta-analysis

Study	Year	Region	Cancer type	No. of cases	Expression	Follow-up time (month)	Detection Method	Survival outcome	NOS score
Barbagallo et al [16]	2019	Italy	glioblastoma	31	Low	≥ 40	RT-qPCR	OS, PFS	6
Cai et al [15]	2019	China	gastric cancer	60	Low	≥ 40	RT-qPCR	OS, DFS	7
Liu et al [17]	2019	China	multiple myeloma	105	Low	≥ 40	RT-qPCR	OS, PFS	6
Lu et al [14]	2020	China	intrahepatic cholangiocarcinoma	92	Low	≥ 60	RT-qPCR	OS	8
Miao et al [18]	2020	China	colorectal cancer	45	Low	≥ 100	RT-qPCR	OS	8
Tong et al [13]	2020	China	non-small cell lung carcinoma	460	Low	≥ 96	RT-qPCR	OS, DFS	8
Yu et al [8]	2018	China	hepatocellular carcinoma	40	Low	≥ 60	RT-qPCR	OS, RFS	8

*DFS* disease-free survival, *OS* overall survival, *PFS* progression-free survival, *RFS* recurrence-free survival, *RT-qPCR* real-time quantitive polymerase chain reaction

### Association between circSMARCA5 expression and prognosis of patients with malignant tumor

Our in-silico analysis using BBCANCER database (http://bbcancer.renlab.org) suggested that circSMARCA5 was downregulated in the EVs of colorectal cancer (logFC = − 0.137), liver cancer (logFC = − 0.27), and pancreatic cancer (logFC = − 0.208), although the differences were not significant (Table S1). We tried to conduct an in-silico analysis on circSMARCA5 using KM plotter database (https://kmplot.com/analysis/). Unfortunately, there was no data concerning circRNAs in the KM plotter database.

**Fig. 3:**
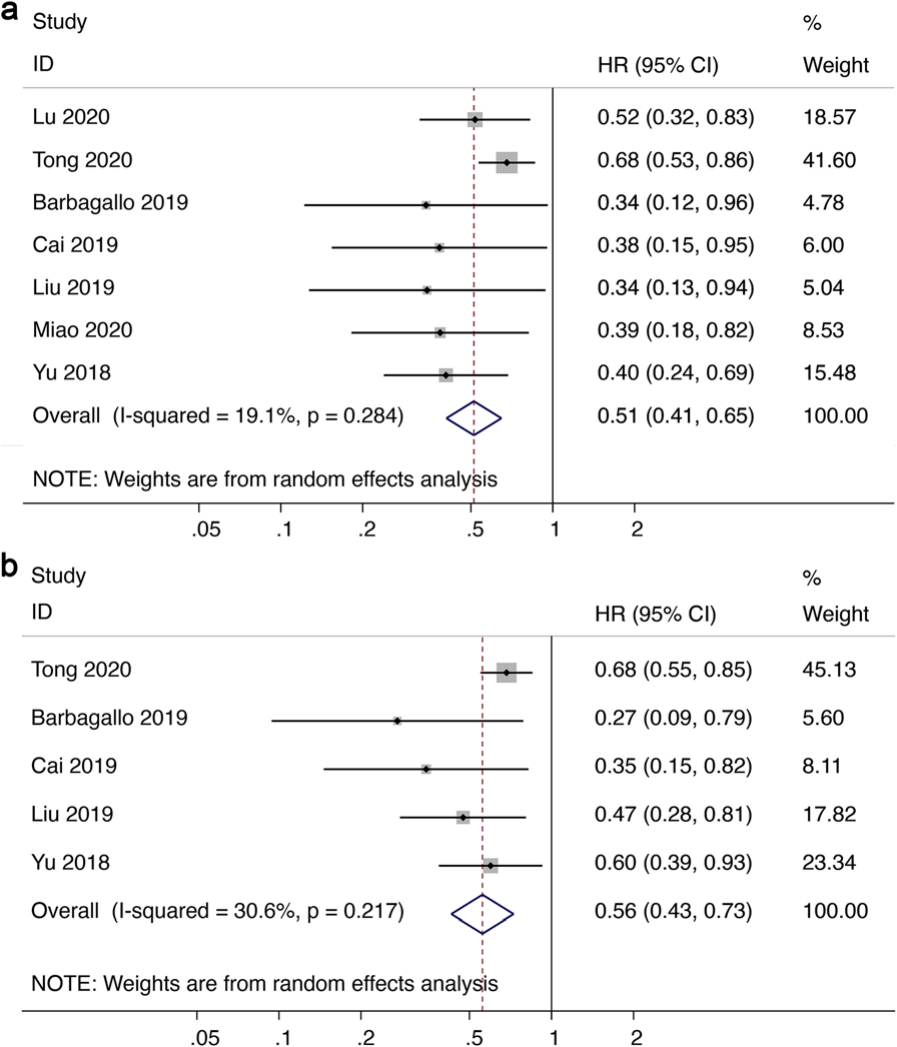
Forest plot of the association between abnormal circSMARCA5 expression and (**a**) OS and (**b**) DFS/RFS/PFS

**Fig. 4:**
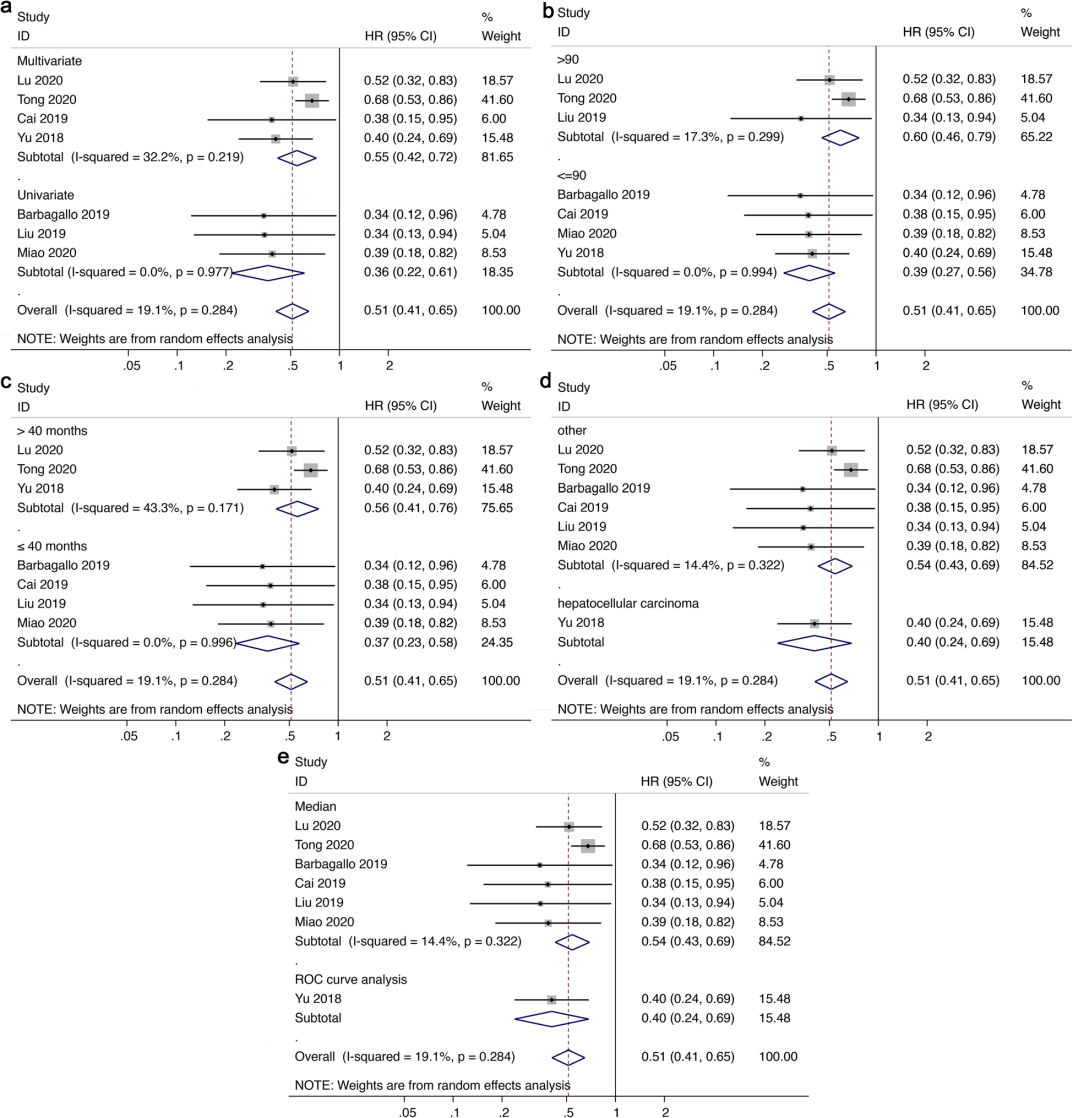
Subgroup analyses of OS, stratified by (**a**) analysis method, (**b**) sample size, (**c**) follow-up time, (**d**) cancer type, (**e**) definition of the cut-off

Meta-analysis of 7 studies involving 833 patients suggested better overall survival (OS) in malignant tumors with high circSMARCA5 expression (HR = 0.51, 95%CI 0.41–0.65, *I*^*2*^ = 19.1%) (Fig. 3a). Consistently, high circSMARCA5 expression was significantly correlated with better disease-free survival (DFS) / recurrence-free survival (RFS) / progression-free survival (PFS) in malignant tumors (HR = 0.56, 95%CI 0.43–0.73, *I*^*2*^ = 30.6%) (Fig. 3b). Results of subgroup analysis by analysis method (Fig. 4a), sample size (Fig. 4b), follow-up time (Fig. 4c), cancer type (Fig. 4d), definition of the cut-off (Fig. 4e) indicated that expression of circSMARCA5 is correlated with better OS in every single subgroup. However, these subgroups have no significant impact on OS.

### Association between circSMARCA5 expression and clinicopathological features of patients with malignant tumor

**Fig. 5:**
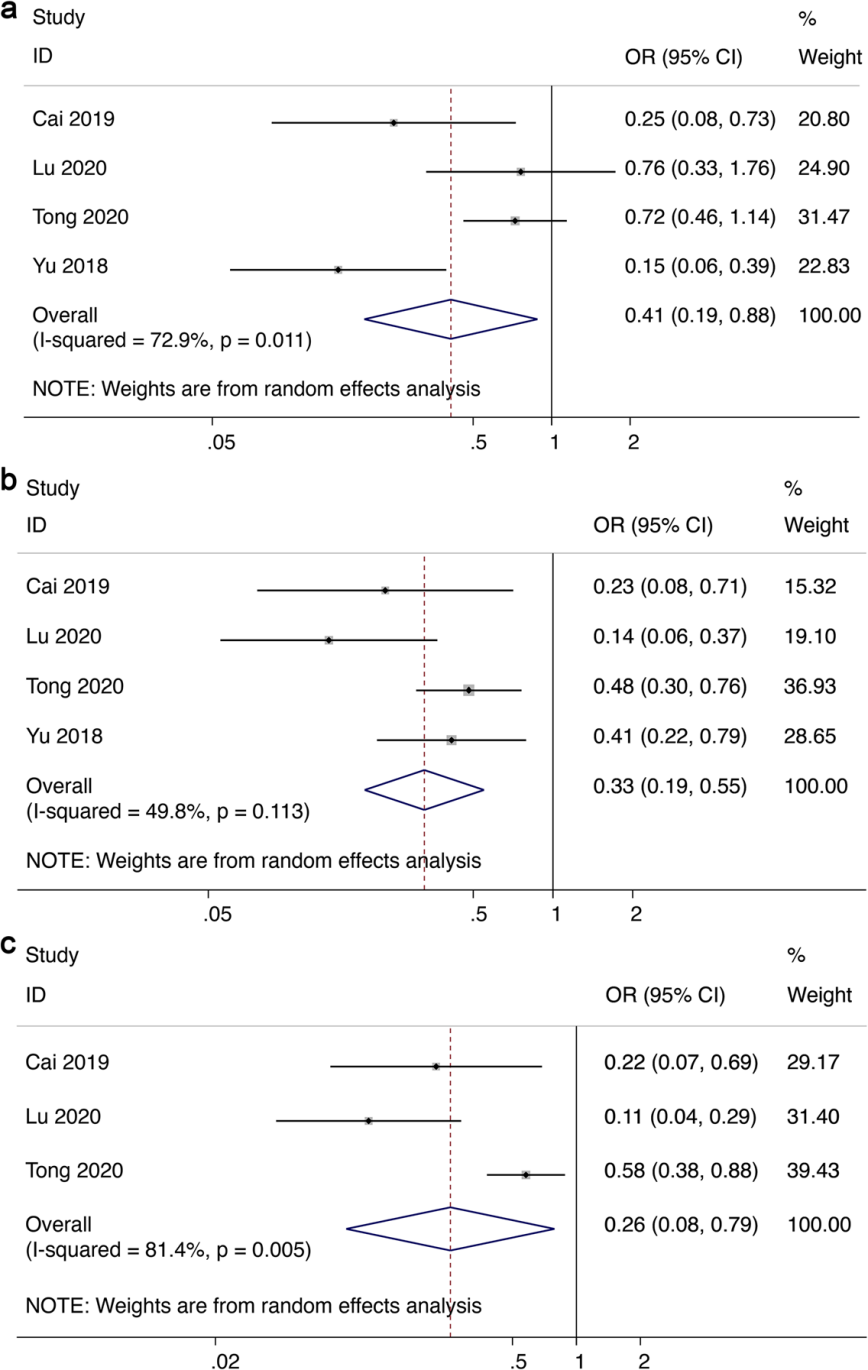
Forest plot of the association between abnormal circSMARCA5 expression and (**a**) differentiation, (**b**) TNM stage, and (**c**) lymph node metastasis

In the pooled analyses of clinicopathological characteristics, malignant tumors with higher circSMARCA5 were better differentiated (OR = 0.41, 95%CI 0.19–0.88, *I*^*2*^ = 72.9%) (Fig. 5a). CircSMARCA5 expression was correlated with less advanced TNM stage (III & IV) (OR = 0.33, 95%CI 0.19–0.55, *I*^*2*^ = 49.8%) (Fig. 5b). In addition, malignant tumors with higher circSMARCA5 expression had less advanced lymph node metastasis (OR = 0.26, 95%CI 0.08–0.79, *I*^*2*^ = 81.4%) (Fig. 5c). It is worth noting that the *I*^*2*^ index showed moderate heterogeniety (> 50%) and as such the different studies may not be comparing the same findings.

### Sensitivity analysis

**Fig. 6:**
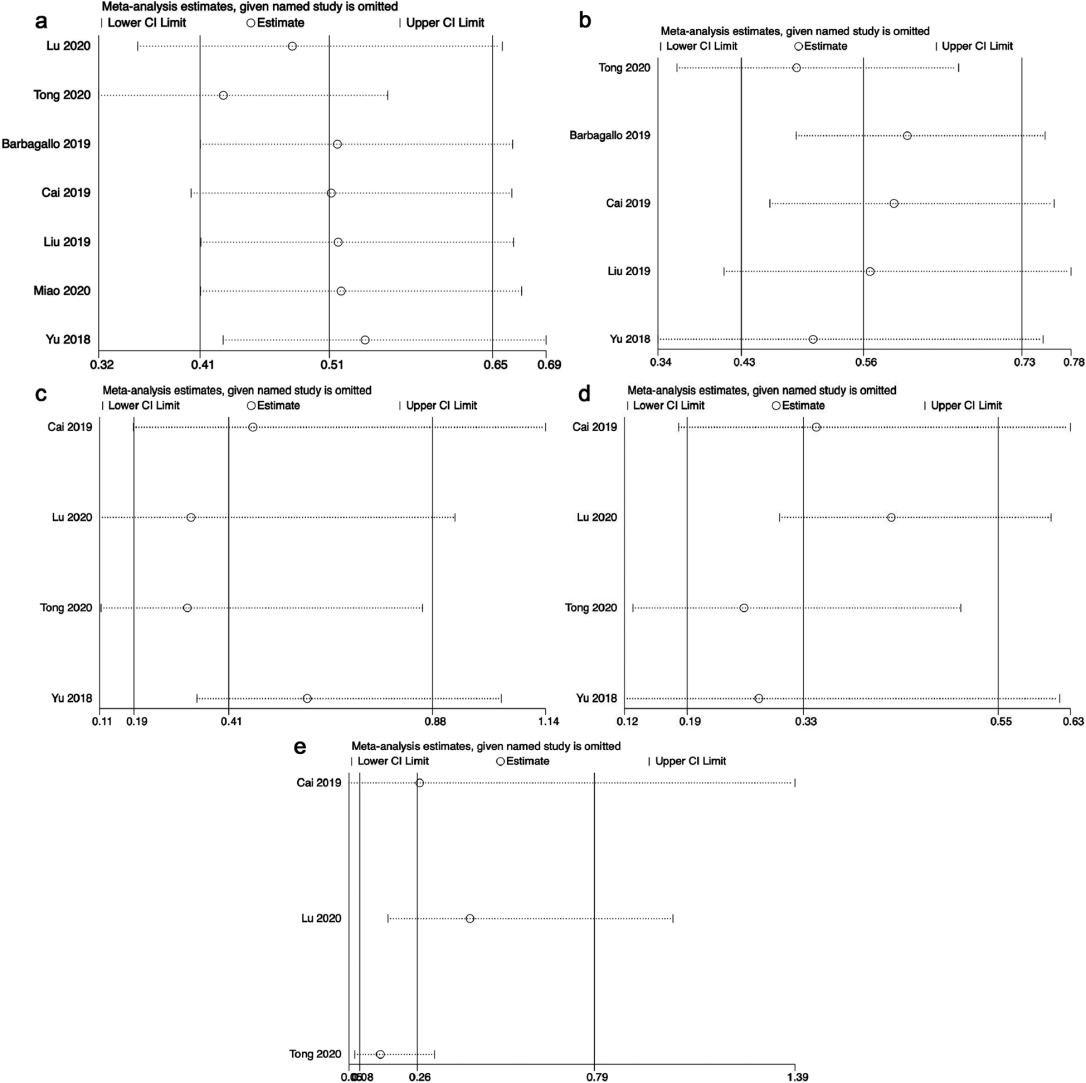
Sensitive analyses of the meta-analyses between abnormal circSMARCA5 expression and (**a**) OS, (**b**) DFS/RFS/PFS, (**c**) differentiation, (**d**) TNM stage, (**e**) lymph node metastasis

Sensitive analyses of the meta-analyses between abnormal circSMARCA5 expression and OS (Fig. 6a), DFS/RFS/PFS (Fig. 6b), differentiation (Fig. 6c), TNM stage (Fig. 6d), and lymph node metastasis (Fig. 6e) suggested that the results of the present meta-analyses were comparatively stable and credible.

## Discussion

Recent studies and meta-analyses have evaluated the role of circRNAs in malignant tumors. For instance, Wang et al validated a novel circRNA as a prognostic factor in acute myeloid leukemia [22]. Han et al constructed a prognostic model of gastric cancer using circRNA-related competitive endogenous network [23]. Yuan et al claimed that circRNAs might serve as potential biomarkers for colorectal cancer [24]. In addition, 2 independent meta-analyses suggested that circRNA CDR1as could act as diagnostic and prognostic biomarkers in various malignant tumors [10, 25]. Exploring the value of circRNAs as biomarkers will provide promising guidance for clinical decision. However, the role of circSMARCA5 in the prognosis of malignant tumors had not been systematically analyzed.

The current meta-analyses, for the first time, assessed the value of circSMARCA5 as a prognostic marker for malignant tumors. In 2017, Kong et al claimed that circSMARCA5 was upregulated in prostate cancer and promoted cell proliferation [26]. Although this is the first publication that reported the biological function of circSMARCA5 in malignant tumor, the value of circSMARCA5 in diagnosis and prognosis was not reported. However, later studies indicated that circSMARCA5 was a tumor suppressor which was downregulated in various malignant tumors. At present, many articles have reported the role of circSMARCA5 in tumors and its relationship with the prognosis of tumor patients. The expression of circSMARCA5 was associated with the survival of non-small cell lung cancer [13], intrahepatic cholangiocarcinoma [14], hepatocellular carcinoma [8], gastric cancer [15], glioblastoma [16] and multiple myeloma [17]. The present meta-analysis systematically summarized the previous studies and assessed circSMARCA5 as a prognostic factor of malignant tumors using detailed search strategy and appropriate inclusion criteria. The studies concerning the association between circSMARCA5 expression and the prognosis of patients with malignant tumors was summarized and systematically analyzed. The association between circSMARCA5 expression and clinicopathological features was also estimated. Results of the meta-analyses suggested that circSMARCA5 expression was associated with survival, differentiation, TNM stage, and lymph node metastasis of patients with malignant tumors. CircSMARCA5 may involve different molecular implications in different types of malignant tumors. For instance, circSMARCA5 could regulate VEGFA mRNA splicing and angiogenesis through the binding of SRSF1 in glioblastoma multiforme [16]. Xu et al reported that circSMARCA5 inhibited DNA damage repair by interacting with its host gene *SMARCA5* [27].

In addition, the pitfalls and limitations of the present study were as follows: (i) Up to now, there are only 7 studies with available data regarding circSMARCA5 in malignant tumors. In different types of malignant tumors, the target of circSMARCA5 may be different. These differences regarding circSMARCA5 in different cancers may induce bias of the meta-analysis. However, circSMARCA5 is indeed related to the prognosis and clinicopathological characteristics of patients with different malignant tumors. Therefore, we enrolled these studies to determined prognostic role of circSMARCA5 in malignant tumours. (ii) Studies on circSMARCA5 were carried out by different research groups, so the methods used were different. The included studies used the ddCT method to analyze the data of RT-qPCR when exploring the expression of circSMARCA5 in cancer and normal tissues. This analysis method that uses GAPDH as an internal reference may be unreliable due to the downregulation of GAPDH in cancer cells [28]. Other internal references may also be dysregulated in cancer cells. Therefore, the use of the dCT method may be better [29].

In 1976, Sanger et al discovered that certain plant RNA viruses exhibited high thermal stability, and for the first time identified a covalently closed circular RNA whose structure was composed of more than one exon [30]. With the continuous advancement of detection technology, more circRNAs have been identified and characterized. CircRNAs played an important role in the tumorigenesis and progression of malignant tumors, including evading growth inhibitors and cell death [8, 31, 32], participating in invasion and metastasis [4, 33], angiogenesis and proliferation signal activation [5, 34]. The circRNA abundance was correlated with cell proliferation in colorectal and ovarian cancer, idiopathic lung fibrosis, and normal human tissues [35]. The negative correlation of circRNA with proliferation may be a general principal in human tissues and not just cancer. They also played a role in regulating tumor signal transduction pathways, including Wnt signal transduction [36], PI3K/AKT [31] and MAPK [32] pathways. CircRNAs are relatively stable and has obvious resistance to ribonuclease (RNase) and other exonucleases due to the lack of 5′ or 3′ ends. Therefore, compared with linear RNA, circRNAs have a longer half-life (> 48 h) [37]. The properties of circRNAs and current researches indicated that they could serve as major regulators of gene expression and had the potential to serve as new biomarkers for malignant tumor and other diseases.

An ideal biomarker should have high sensitivity, specificity and predictive efficacy. Although research on circRNAs has just started, some features of circRNA suggest that they may be potentially valuable biomarkers for malignant tumors and other diseases. First, they are usually expressed in a specific way in tissues and developmental stages; second, the lack of 5′ or 3′ ends makes circRNA highly resistant to RNase activity. In addition, they are abundantly expressed in various body fluids and tissues, including plasma, serum, blood, and even in exosomes, making them ideal biomarkers for liquid biopsy. Therefore, circular RNAs could serve as stable biomarkers [9, 38]. Previous meta-analyses concerning the correlation between circRNAs indicated that the abnormal expression of CDR1as [10, 25] or circHIPK3 [11] was associated with prognosis and clinicopathological characteristics of human malignant tumors. In general, circRNA has great potential as cancer biomarkers and even new therapeutic molecules, but the research on these molecules is still at a preliminary stage. The precise functions of most circRNAs and the specific mechanisms of action in diseases are still unclear.

## Conclusions

In conclusion, the present study suggested that circSMARCA5 has a remarkable correlation between its aberrant expression and prognosis as well as clinicopathological features of patients with malignant tumors. CircSMARCA5 is a promising biomarker with high efficiency which may become an ideal indicator for malignant tumors in the future.

## Supplementary Information

**Additional file 1: Table S1** The differential expression of circSMARCA5 in colorectal cancer, liver cancer, and pancreatic cancer.

## Data Availability

The datasets used and/or analyzed during the current study are available from the corresponding author on reasonable request.

## Abbreviations

CircRNA: circular RNA
CI: confidence interval
DFS: disease-free survival
HR: hazard ratio
NOS: Newcastle-Ottawa Score
OR: odds ratio
OS: overall survival
PFS: progression-free survival
RFS: recurrence-free survival
RT-qPCR: real-time quantitive polymerase chain reaction
RNA: ribonucleic acid
SMARCA5: SWI/SNF related, matrix associated, actin dependent regulator of chromatin, subfamily a, member 5
