# Supplementary material for: Circular RNA circSMARCA5 is a prognostic biomarker in patients with malignant tumor: a meta-analysis
Source: BMC Cancer. 2021 May 25;21:600. doi: 10.1186/s12885-021-08316-3 (PMC8145840; doi:10.1186/s12885-021-08316-3)
Supplement: Supplementary file 1 — Additional file 1: Table S1 The differential expression of circSMARCA5 in colorectal cancer, liver cancer, and pancreatic cancer. [file 12885_2021_8316_MOESM1_ESM.docx]

Table S1 The differential expression of circSMARCA5 in colorectal cancer, liver cancer, and pancreatic cancer.

| Analysis ID | Tumor | Tissue | Design | Sample Number | Gene | logFC | Average Expression | P-value | Adjusted P-value |
| --- | --- | --- | --- | --- | --- | --- | --- | --- | --- |
| CC_R3 | Colorectal cancer | EVs | Tumor-Normal | 18(12-6) | circ_028245 | -0.137 | 542.42 | 0.574 | 0.8254 |
| CC_T4 | Colorectal cancer | Tissue | Tumor-Normal | 85(20-65) | circ_028245 | -0.203 | 6.0154 | 0.6966 | 0.9914 |
| LI_R1 | Liver cancer | EVs | Tumor-Normal | 27(21-6) | circ_028245 | -0.27 | 530.6 | 0.1066 | 0.2789 |
| PC_R1 | Pancreatic cancer | EVs | Tumor-Normal | 20(14-6) | circ_028245 | -0.208 | 624.11 | 0.4168 | 0.7694 |

Data were retrieved from the BBCANCER database.
